# Analysis of consumer purchase intentions using functional near-infrared spectroscopy(fNIRS): A neuromarketing study on the aesthetic packaging of Korean red ginseng products

**DOI:** 10.1371/journal.pone.0326213

**Published:** 2025-06-17

**Authors:** SuJin Bak, Jaeyoung Shin, Daeun Kim, Sungkean Kim

**Affiliations:** 1 Advanced Institute of Convergence Technology, Suwon, Gyeonggi-do, South Korea; 2 Department of AI Data Engineering, Korea National University of Transportation, Uiwang, Gyeonggi-do, South Korea; 3 Department of Applied Artificial Intelligence, Hanyang University, Ansan, Gyeonggi-do, South Korea; 4 Department of Human-Computer Interaction, Hanyang University, Ansan, Gyeonggi-do, South Korea; National University of Sciences and Technology, PAKISTAN

## Abstract

**Purpose:**

This study investigates the consumer behavior patterns for Korean red ginseng products using functional near-infrared spectroscopy (fNIRS), a neuroimaging device. By measuring brain activities, we aim to develop neuromarketing techniques and provide insights into consumer purchasing decisions influenced by the aesthetic features of product packaging.

**Methods:**

Using an fNIRS device, we measured the hemodynamic responses of 50 healthy participants within the prefrontal cortex (PFC) during virtual purchase-response paradigm experiments. These experiments consisted of two scenarios depending on the purpose of purchasing red ginseng products; purchasing for self-consumption and purchasing as gifts for others. These two purchase scenarios were designed to elucidate the relationship between two purchasing scenarios and aesthetic product packaging.

**Results:**

We observed distinct differences in purchasing behavior between the two scenarios influenced by the packaging of red ginseng products. We found significantly higher prefrontal ∆HbO activation during the gift-giving condition (Task 1) compared to self-consumption (Task 2). Survey results also indicated greater purchase intention (M = 4.35) and great packaging satisfaction (M = 4.18) in Task 1 (*p* < 0.01) compared to Task 2. No significant differences were observed across gender or age groups. These findings suggest that packaging has a differentiated effect depending on the purchasing context.

**Conclusions:**

Our findings highlight the need for customized marketing strategies while proposing separate packaging designs for self-consumption and gifts. This study demonstrates the potential of neuromarketing technology based on fNIRS in predicting consumer behavior and improving marketing strategies.

**Significance:**

Understanding the neural mechanisms of purchasing decisions enables Korean health functional food companies to align their marketing strategies with consumer preferences, thereby enhancing both sales performance and customer satisfaction.

## 1. Introduction

With the growing global emphasis on health and wellness [1], the health food market has experienced significant expansion, particularly in demand for products like Korean red ginseng, a hallmark of traditional Korean health foods. Red ginseng has gained popularity across various demographics, expanding its appeal from older generations to younger consumers. This shift is largely attributed to rebranding efforts and heightened health consciousness among younger populations, resulting in a growing demand for red ginseng in both domestic and international markets [2–4]. Despite its growing appeal, the impact of aesthetic packaging on consumer purchasing decisions, especially for red ginseng products, remains insufficiently explored.

Packaging has long been recognized as a pivotal factor in shaping consumer perceptions and influencing decision-making processes at the point of sale [5]. While prior research has predominantly focused on the functional aspects of packaging—such as protection, storage, and hygiene—the specific impact of aesthetic elements, including color, design, and luxury features, on consumer behavior has been less comprehensively examined. In particular, comparative studies on packaging preferences for self-consumption versus gifting remain relatively limited, with only a few investigations addressing this distinction systematically. In particular, it is considered that business officials and consumers implicitly spend more time and money on gift products for others, and tend to wrap them in a luxurious and elaborate manner, but this has not been scientifically revealed. Furthermore, existing marketing studies have examined consumer behavior using data obtained from self-reported measurements, which mostly depend on people’s memories of their own experiences. These studies must subjectively rely on the people’s feelings and thoughts, which are not absolute [6].

Nevertheless, existing studies provide valuable insights into related dynamics. For instance, previous research suggests that the degree of intimacy between gift givers and recipients significantly influences packaging choices [7]. Another study found that gift packaging affects the recipient’s perception, providing insights into the giver’s thoughts and efforts [8]. Luxurious and thoughtfully designed packaging has been shown to effectively convey the giver’s care and effort, enhancing the perceived value of the gift [9]. Meanwhile, younger consumers increasingly prioritize self-consumption when purchasing products like red ginseng, viewing such purchases as “gifts to themselves” [10]. This emerging trend reflects a shift toward self-oriented consumption, emphasizing personal preferences and needs over traditional gift-giving practices [11].

To address these gaps, this study employs functional near-infrared spectroscopy (fNIRS), a non-invasive neuroimaging tool [12–16], to analyze the cognitive and emotional responses of consumers during purchasing decisions. Unlike traditional self-report methods, which rely heavily on subjective memories and perceptions, fNIRS provides real-time measurements of brain activity, offering deeper insights into unconscious consumer behavior [17]. Specifically, fNIRS measures changes in oxygenated hemoglobin (∆HbO) and deoxygenated hemoglobin (∆HbR) in the prefrontal cortex (PFC), a region associated with decision-making, planning, and cognitive control [18–20]. Recent advancements in neuromarketing have demonstrated the utility of fNIRS in identifying brain mechanisms underlying consumer behavior and decision-making [21,22].

Therefore, we propose a neuromarketing methodology to predict consumers’ purchase purposes for Korean red ginseng gift sets using fNIRS, the latest neuroimaging modality. To accomplish this goal, we compared the hemodynamic responses of fNIRS observed in Session 1, which elicited consumer behavior in purchasing gift red ginseng products for others, and Session 2, which elicited consumer behavior in purchasing red ginseng products for self-consumption. We also analyzed consumer behavior using virtual purchasing decisions and response times of red ginseng products on the market. Then, purchase intentions for price, preference, and packaging status were classified into a 5-point Likert scale using a self-report measurement method. As a result, we found statistically significant fNIRS signal differences between Sessions 1 and 2 in consumer behavior focused on red ginseng purchases. These empirical findings are consistent with the self-reported responses. This study provides promising results for neuromarketing technology by measuring brain activities with fNIRS to enhance understanding of consumer behavior patterns for Korean red ginseng products.

## 2. Materials and methods

### 2.1. Participants

Fifty healthy people (ages: 24.2±3.3 years; 22 females) participated in this study. All participants were actively recruited over the course of approximately one month, from June 2 to June 28, 2024. We recorded the demographics of the participants in Table 1. All participants had normal or corrected-to-normal vision. The participants had no history of medical, mental, or psychological disorders. This study utilized data from all 50 participants, representing the entire research population. All participants provided written informed consent and were compensated for their participation. This study was conducted following the ethical guidelines established by the Institutional Review Board of Wonkwang University (No. WKIRB–202405–HR–023) and the Declaration of Helsinki (World Medical Association, 2013).

**Table 1:** Demographic information summary.

Variable	Category	Number of subjects	Percentage (%)
**Race**	Asian	50	100
**Gender**	Males	28	56
	Females	22	44
**Age range (mean** ± **std.)**	20-30’s	24.2±3.3	100
**Employment**	Not employed	3	6
	Student	45	90
	Employed	2	4
**Education**	A high school graduate	36	72
	Bachelor’s degree	14	28
**Main hand**	Right	47	94
	Left	2	4
	Both	1	2

### 2.2. fNIRS device setting and signal processing

**Fig 1:**
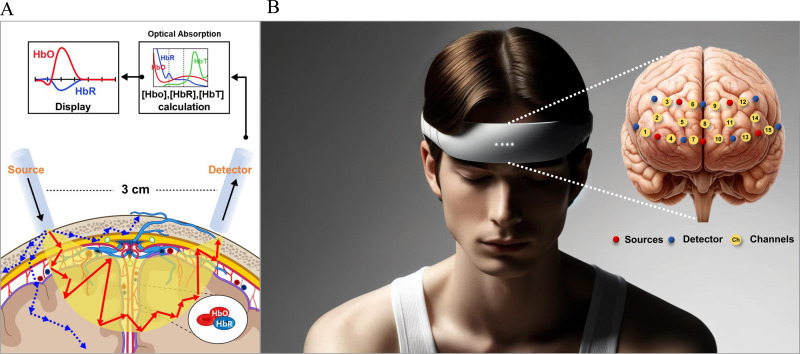
Concepts of fNIRS (A) [ 22] and the arrangement of optodes (B) in the NIRSIT Lite systems.

fNIRS signals can interpret hemodynamic activation patterns in the PFC when performing our experimental tasks. To measure the brain’s hemodynamic responses in the PFC, we used an fNIRS device (NIRSIT Lite, OBELAB Inc., Korea). To provide detailed guidance on the specifications of fNIRS, Fig 1 illustrates the fNIRS concepts (A) [22] and channel configuration (B).

In Fig 1A, the detector captured light scattered through tissue, using model of light propagation. The light could penetrate approximately 8 mm into the cerebral cortex while maintaining a distance of 3 cm between the source and detector. The fNIRS device reflected the absorption properties of living tissues to measure changes in the local concentrations of oxy-hemoglobin (HbO) and deoxy-hemoglobin (HbR) within the crescent-shaped near-infrared region through the skull [23,24]. The crescent-shaped paths represented the near-infrared light (NIR) photons’ traveling area, while the blue dotted arrows represent light scattering. The red-colored arrows showed the distance traveled by photons, which is corrected by the differential path length factor. Consequently, fNIRS could quantitatively measure the hemodynamic changes by absorbing near-infrared rays into the scalp and measuring the emitted light.

Fig 1B shows that the fNIRS device has a total of 15 yellow channels composed of 5 sources (the red circles) and 7 detectors (the blue circles) covering the forehead and an example of source-detector pairs in detail. We recorded the optical density data at a frequency of 8.138 Hz and configured it to detect hemodynamic activity at wavelengths of 780 nm and 850 nm. All signals were converted to oxygenated and deoxygenated concentration changes using the modified Beer–Lambert law [25]. To obtain the optical density data, we adopted prior research [26]. Measured fNIRS signals were digitally bandpass filtered using a zero-phase filter based on a fifth-order Butterworth filter within the range of 0.005–0.1 Hz to eliminate possible physiological noise. Filtered data were segmented into epochs ranging from −5–60 s relative to the task onset (0 s). The epoch was subjected to a baseline correction to subtract the mean value within a reference interval from −5–0 s. The temporal means of the fNIRS data in each channel were calculated by averaging the fNIRS data from the start to the end time (0–60 s) in each epoch.

### 2.3. Experimental procedures

To investigate brain activation patterns, we designed virtual two purchase-decision tasks depending on the Session 1 and Session 2 paradigms using E-Prime 3.0. All subjects were asked to decide on purchasing displayed red ginseng products under Session 1 (including for gifts to others) and Session 2 (including for self-consumption). Each experimental task comprised 10 trials. In each task, the participants are free to choose the ginseng product they want. The selected Korean red ginseng brand was Jung-Kwan-Jang [27], known as the Korea ginseng corporation (KGC)’s red ginseng health food brand. The Korean red ginseng product lines are randomly divided into two major types (i.e., Branded packaging, and Unlabeled package types), which were displayed on the screen. All participants were free to purchase one of their choices from the four products displayed on the screen. Each product was presented randomly during the experiment. One session lasted for 10 minutes and included the following stages (trial number display (0.5 s), task (24 s), rest (35 s)). The order of the sessions was randomized and counterbalanced. Then, all participants completed a self-reported measurement using Google Form, which is a popular survey platform, before and after the experiment.

### 2.4. Survey measurement

In this study, multiple-item scales were used to measure Korean red ginseng buying behavior. The items in the self-reported measurements were designed based on a review of the following literature: “Are you willing to purchase the product for (i.e., for gift to others or for self-consumption) even if there is a price difference in according to the packaging?”, “Are you satisfied with the Korean red ginseng?”, and “Is packaging important to you when purchasing red ginseng?”. All respondents answered these items. For the manipulation check of price, preference, and package status, all items were measured using a modified multi-item scale developed by Refs.[28] and [29]. All items were used on a 5-point Likert scale ranging from ‘strongly disagree’ to ‘strongly agree’.

Also, we investigated the response differences in product appearance and purchase price according to product beneficiaries. To this end, the participants were asked about their product preferences concerning the high priced and high-end appearance of the product based on the intended beneficiaries. It was divided into Tasks 1 and 2; 1) Despite the high cost and limited funding, would you still like to buy a luxurious and branded packed red ginseng gift set for your valued acquaintance until you receive your next allowance or salary? 2) Despite the high cost and limited funding, would you still like to buy a luxurious and branded packed red ginseng gift set for yourself until you receive your next allowance or salary?

### 2.5. Behavioral analysis

Consumers’ purchase decision-making is often described as a rational process of selecting the best option from various alternatives. During this process, individuals often dedicate significant resources to enhancing the visual expressiveness of gift packaging [30], so it may take a considerable amount of time to determine which product to buy [31].

Based on these prior studies, we investigated and compared the buying tendency for red ginseng products between Session 1 (i.e., for gifting to others) and Session 2 (i.e., for self-consumption) by calculating the sum of the unlabeled package products virtually purchased by the participants during 30 attempts. We also measured the reaction time for purchasing decisions.

### 2.6. Statistical analysis

All statistical analyses were performed using the SPSS, version 26 (SPSS Inc., Chicago, IL, USA). Variables were examined for normality, mean (M), standard deviation (s.d.). We conducted statistical analyses of fNIRS signal values and self-report measurement responses. Levene’s test was conducted to determine the homogeneity of variance, and then we used a paired sample t-test with a significance threshold of p < 0.05 to analyze the fNIRS signal differences between Sessions 1 and 2. To enhance the reliability and generalizability of this study, we employed both t-test and one-way analysis of variance (ANOVA) to analyse differences in survey responses according to participants’ gender and age. A Scheffe correction was employed to control for Type I error in multiple comparisons involving gender- and age-related differences, and the results still yielded a p-value less than 0.05.

Furthermore, we manipulated the price (i.e., high or low conditions), product preference (i.e., high or low), and packaging status (i.e., for gifts or for self-consumption), and then calculated the self-reported score differences using an independent t-test. To assess the internal consistency of self-reported measurements, we calculated the Cronbach’s alpha (α). The value of α ranges between 0 and 1, with higher values indicating greater reliability of the self-reported measurements.

## 3. Results

### 3.1. Subject behavioral analyses: Buying decisions and response time

We assessed whether the participants chose to buy the product and recorded the time they took to decide on the red ginseng product package. All participants chose one red ginseng product from four products.

A t-test was conducted with red ginseng product buying behavior as a dependent variable and online shopping (for gift to others vs. for self-consumptions) as an independent variable. Table 2 shows the products that all subjects decided to purchase and their average values. Buying behavior in Session 1 (M = 0.17, s.d. = 0.24) is lower than Session 2 (M = 0.54, s.d. = 0.27); This result indicated that participants purchased branded packaged products more frequently in Session 1 than in Session 2. Except for 11 out of 50 subjects, all participants showed statistically significant differences in purchasing outcomes between the two sessions.

**Table 2:** Statistical results: buying decision responses.

No.	N = 30				*t(p)*		No.	N = 30				*t(p)*	
	mean		std.					mean		Std.			
	S1	S2	S1	S2				S1	S2	S1	S2		
P1	0.000	0.100	0.000	0.305	*−1.795(0.083)*	*N.S.*	P26	0.000	0.130	0.000	0.346	−2.112(0.043) ^*^	
P2	0.000	0.230	0.000	0.430	*−2.971(0.006)* ^**^		P27	0.130	1.000	0.346	0.000	−13.730(0.000) ^***^	
P3	0.070	0.230	0.254	0.430	*−1.828(0.074)*	*N.S.*	P28	0.000	0.000	0.000	0.000	^*1)*^	
P4	0.000	0.200	0.000	0.407	*−2.693(0.012)* ^*^		P29	0.670	0.070	0.479	0.254	6.058(0.000) ^***^	
P5	0.030	0.370	0.183	0.490	*−3.491(0.001)* ^**^		P30	0.100	0.070	0.305	0.254	0.460(0.647)	*N.S.*
P6	0.030	0.030	0.183	0.183	*0.000(1.000)*	*N.S.*	P31	0.070	0.930	0.254	0.254	−13.230(0.000) ^***^	
P7	0.230	0.730	0.430	0.450	*−4.400(0.000)* ^***^		P32	0.800	0.400	0.407	0.498	3.406(0.001) ^**^	
P8	0.000	0.900	0.000	0.305	*−16.155(0.000)* ^***^		P33	0.000	0.330	0.479	0.088	−3.808(0.001) ^**^	
P9	0.200	0.570	0.407	0.504	*−3.101(0.003)* ^**^		P34	0.500	1.000	0.509	0.000	−5.385(0.000) ^***^	
P10	0.030	0.400	0.183	0.498	*−3.785(0.001)* ^**^		P35	0.470	0.970	0.507	0.183	−5.078(0.000) ^***^	
P11	0.030	0.230	0.183	0.430	*−2.344(0.024)* ^*^		P36	0.000	0.230	0.000	0.430	−2.971(0.006) ^**^	
P12	0.000	0.930	0.000	0.254	*−20.149(0.000)* ^***^		P37	0.030	0.930	0.183	0.254	−15.771(0.000) ^***^	
P13	0.030	0.630	0.183	0.490	*−6.283(0.000)* ^***^		P38	0.330	0.070	0.479	0.254	2.693(0.010) ^*^	
P14	0.030	0.030	0.183	0.183	*0.000(1.000)*	*N.S.*	P39	0.130	0.630	0.346	0.490	−4.566(0.000) ^***^	
P15	0.000	0.230	0.000	0.430	*−2.971(0.006)* ^**^		P40	0.000	0.700	0.000	0.466	−8.226(0.000) ^***^	
P16	0.000	0.970	0.000	0.183	*−29.000(0.000)* ^***^		P41	0.170	0.600	0.379	0.498	−3.791(0.000) ^***^	
P17	0.030	0.530	0.183	0.507	*−5.078(0.000)* ^***^		P42	0.000	1.000	0.000	0.000	^*1)*^	
P18	0.130	0.600	0.346	0.498	*−4.215(0.000)* ^***^		P43	0.000	0.930	0.000	0.254	−20.149(0.000) ^***^	
P19	0.030	0.970	0.183	0.183	*−19.799(0.000)* ^***^		P44	0.030	1.000	0.183	0.000	−29.000(0.000) ^***^	
P20	0.030	1.000	0.183	0.000	*−29.000(0.000)* ^***^		P45	1.000	1.000	0.000	0.000	^*1)*^	
P21	1.000	0.000	0.000	0.000	^*1)*^		P46	0.370	0.030	0.490	0.183	3.491(0.001) ^**^	
P22	0.800	1.000	0.407	0.000	*−2.693(0.012)* ^*^		P47	0.000	1.000	0.000	0.030	^*1)*^	
P23	0.400	0.000	0.498	0.000	*4.397(0.000)* ^***^		P48	0.030	0.570	0.183	0.504	−5.449(0.000) ^***^	
P24	0.070	0.030	0.254	0.183	*0.584(0.561)*	*N.S.*	P49	0.000	0.500	0.000	0.509	0.379(0.000) ^***^	
P25	0.630	1.000	0.490	0.000	*−4.097(0.000)* ^***^		P50	0.000	0.830	0.000	0.379	−12.042(0.000) ^***^	
Overall S1 M ± SD							^*2)*^0.173 ± 0.238						
Overall S2 M ± SD							0.537 ± 0.274						

* p < .05, ** p < .01, *** p < .001, N.S. Not Significant.

1) T-test could not be conducted due to the absence of variability in the data.

2) These response scores indicate that the respondent decided to buy a red ginseng product with great packaging as it approached zero.

Furthermore, Table 3 shows the response time and the average time taken by the subject to decide on a purchase. Buying behavior in Session 1 (M = 3,519 s, s.d. = 1,320 s) is lower than Session 2 (M = 3,253 s, s.d. = 1,155 s). This indicated that participants took more time to choose products in Session 1 compared to Session 2. It showed a statistically significant difference in response time between the two sessions for all subjects, except for 23 out of 50 participants.

**Table 3:** Statistical results: response time to buying decision behaviors.

No.	N = 30 (seconds)				*t(p)*		No.	N = 30 (seconds)				*t(p)*	
	M		s.d.					M (s)		s.d.			
	S1	S2	S1	S2				S1	S2	S1	S2		
P1	2864	3394	1688	1252	*−1.603(0.120)*	*N.S.*	P26	3976	4019	1207	1557	*−0.131(0.897)*	*N.S.*
P2	3507	3682	1757	1959	*−0.336(0.740)*	*N.S.*	P27	4631	2567	1609	717	*6.597(0.000)* ^***^	
P3	3670	2792	1655	1387	*1.946(0.061)*	*N.S.*	P28	3850	3697	1222	1053	*0.587(0.562)*	*N.S.*
P4	2995	2555	1486	1387	*1.181(0.247)*	*N.S.*	P29	2913	3670	760	851	*−4.019(0.000)* ^***^	
P5	3431	2464	1698	628	*2.729(0.011)* ^*^		P30	3528	4843	1584	1318	*−3.513(0.001)* ^**^	
P6	1976	2191	1027	909	*−0.808(0.425)*	*N.S.*	P31	5321	3139	1539	1805	*5.195(0.000)* ^***^	
P7	3545	2112	875	400	*7.481(0.000)* ^***^		P32	3325	3810	1801	2275	*−1.033(0.310)*	*N.S.*
P8	5110	2280	1501	1069	*10.852(0.000)* ^***^		P33	4093	4566	1521	1186	*−1.336(0.192)*	*N.S.*
P9	4459	4596	1191	1051	*−0.590(0.560)*	*N.S.*	P34	3006	2125	774	721	*4.124(0.000)* ^***^	
P10	4841	5163	1870	1575	*−0.658(0.516)*	*N.S.*	P35	2849	2825	927	1435	*0.094(0.926)*	*N.S.*
P11	3626	4019	1398	1791	*−0.933(0.359)*	*N.S.*	P36	3422	4927	1081	1149	*−4.724(0.000)* ^***^	
P12	3640	1984	1999	1368	*3.643(0.001)* ^**^		P37	663	2033	1082	1445	*5.283(0.000)* ^***^	
P13	3212	5081	1310	1324	*−4.835(0.000)* ^***^		P38	3461	4565	1473	1776	*−2.892(0.007)* ^**^	
P14	3664	4074	916	663	*−2.092(0.045)* ^*^		P39	2884	4320	1165	1546	*−4.614(0.000)* ^***^	
P15	3429	3462	1267	1580	*−0.097(0.924)*	*N.S.*	P40	4055	4481	1114	956	*−1.361(0.184)*	*N.S.*
P16	3973	1233	1871	448	*7.691(0.000)* ^***^		P41	3170	3240	1106	1229	*−0.253(0.802)*	*N.S.*
P17	3249	4498	865	1193	*−4.623(0.000)* ^***^		P42	4286	2933	1336	1684	*4.208(0.000)* ^***^	
P18	4522	4202	2496	2271	*0.555(0.583)*	*N.S.*	P43	2357	2554	2825	1367	*−0.324(0.748)*	*N.S.*
P19	4224	1404	1034	258	*14.635(0.000)* ^***^		P44	4738	1118	1410	341	*14.126(0.000)* ^***^	
P20	3224	1671	1301	749	*5.412(0.000)* ^***^		P45	2342	1763	1038	626	*2.675(0.012)* ^*^	
P21	2665	1359	785	417	*8.515(0.000)* ^***^		P46	3057	3850	694	1150	*−3.968(0.000)* ^***^	
P22	3552	1351	1413	415	*8.441(0.000)* ^***^		P47	4164	3710	1454	1294	*1.305(0.202)*	*N.S.*
P23	3872	4406	1119	1582	*−1515(0.141)*	*N.S.*	P48	3206	3062	665	706	*0.813(0.423)*	*N.S.*
P24	4490	5267	1895	1088	*−2.054(0.049)* ^*^		P49	3336	3210	687	0.737	*0.737(0.467)*	*N.S.*
P25	1820	2672	636	1172	*−3.433(0.002)* ^**^		P50	3794	3751	901	894894	*0.210(0.835)*	*N.S.*
**Overall S1 M** ± **SD**							3,519 ± 1,320 (s)						
**Overall S2 M** ± **SD**							3,253 ± 1,155 (s)						

* p < .05, ** p < .01, *** p < .001, N.S. Not Significant.

### 3.2. Analysis of survey responses: The effect of product appearance on perceived value by others

**Fig 2:**
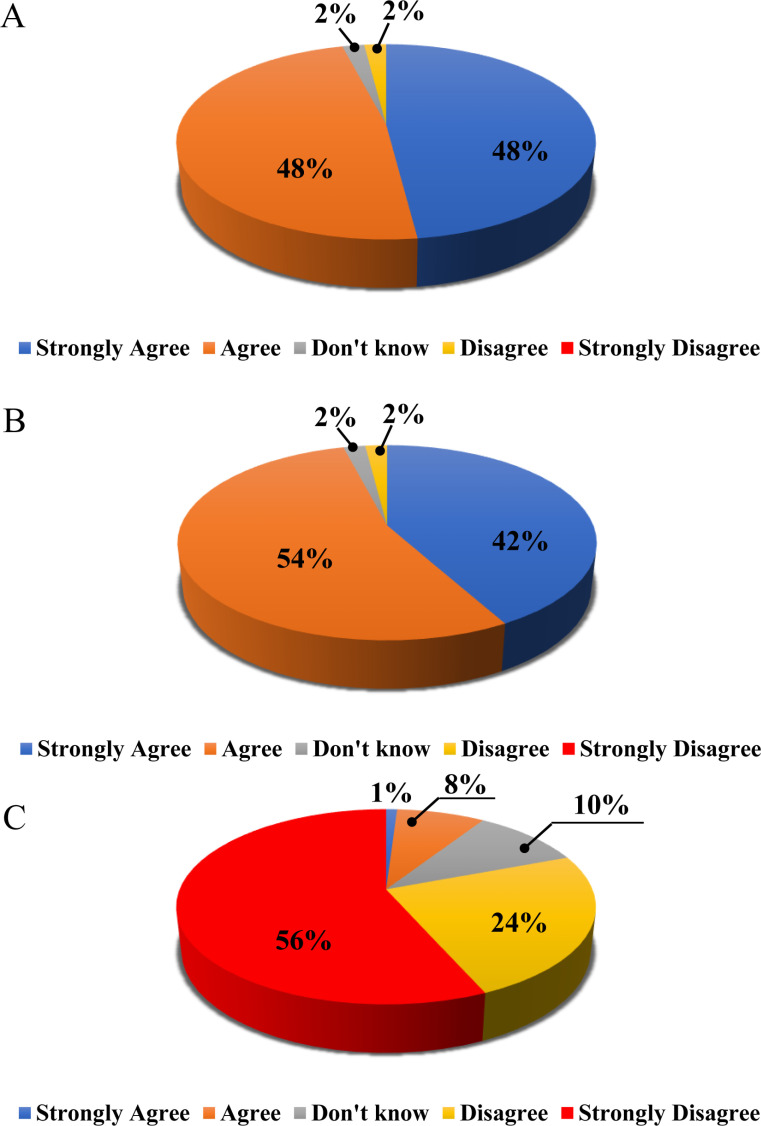
Survey results illustrating perceptions of gift packaging and its influence on social interactions: (A) Participant responses regarding the impact of gift packaging on social interaction, (B) Results from Task 1 based on 50 survey responses, and (C) Results from Task 2 derived from the same participant group.

We surveyed fifty participants in this experiment using Google Forms. We asked them the question, “Do you think the value or appearance of a gift is one of the measures by which the other person judges me?” The results of the responses from fifty people were shown graphically in Fig 2A. Out of the fifty responses, 48% answered “Strongly Agree,” and another 48% answered “Agree,” indicating that 96% of the participants answered that it is a measure of how the other person judges them through a gift. These findings indicated that the giver’s affection for the recipient is expressed through the gift, emphasizing the significance of thoughtful selection and packaging.

### 3.3. Analysis of survey responses: The impact of high prices and luxury packaging based on beneficiaries

Fig 2B and C showed the fifty responses to the two scenarios in graphs, respectively. Fig 2B represented the outcome of Task 1, while Fig 2C illustrated Task 2. As a result, 42% of respondents viewed luxury packaging positively despite its high cost, with an additional 54% indicating they would still make the purchase in Task 1. On the other hand, 56% of respondents stated they would not be inclined to purchase expensive packaged products for themselves, with an additional 24% affirming they would also refrain from buying them in Task 2. The survey results indicated a statistically significant difference in respondents’ answers between the two tasks (p<0.001***). Ultimately, purchase intentions differed based on the gift recipient, with varying results influenced by the price differences between branded and unlabeled packaged products.

### 3.4. Manipulation check and survey findings

In this study, the reliability of survey was validated with Cronbach’s alpha coefficients above 0.6 (α) by calculating packaging-dependent pricing (α = 0.633) and packaging-dependent consumer emotions (α = 0.606). Furthermore, we compared gender differences in participants’ preferences for Task 1 and Task 2 using independent *t*-tests. The results showed that the p-value for Task 1 was 0.925, and for Task 2 it was 0.067, indicating no statistically significant gender differences in either task. Similarly, all participants were divided into age groups according to birth year (before/after 2000) and then, we compared survey responses to Task 1 and Task 2. The results showed no statistically significant differences in preferences for either task across the age groups.

These results ensure the reliability and generalizability of survey responses for two tasks. Participants reported a significant difference between two variable values (***p < 0.01), which suggested that there was a difference in product purchase price depending on the beneficiary types (i.e., others or themselves). Participants indicated that they were more likely to purchase red ginseng to give as a gift to others in Task 1 (M = 4.35, s.d. = 0.09) than to purchase red ginseng for self-consumption in Task 2 (M = 1.75, s.d. = 0.15; F = 10.408, *p < 0.01), even if packaging costs were high.

We also showed that packaging-dependent consumer emotion variables differed between two tasks (***p < 0.01). Participants perceived that the positive purchase satisfaction in Task 1 with buying red ginseng for gifts to others was greater (M = 4.18, s.d. = 0.11) than that of Task 2 with buying red ginseng for self-consumption (M = 3.53, s.d. = 0.15; F = 8.838, *p < 0.01). Thus, these results suggested that packaging should be tailored separately for self-consumption and gift to others in the domestic red ginseng sales strategy.

### 3.5. Comparison of two sessions in brain activities

**Fig 3:**
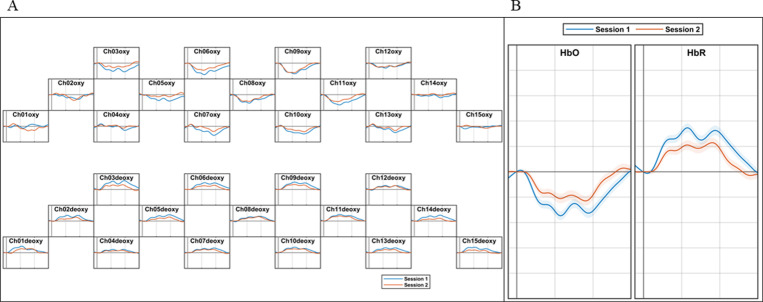
Grand averages across 15 channels of all participants (A) and averaged hemodynamic signals (B) of temporal concentration changes of ΔHbO/HbRs within the Session intervals. The gray line marks the beginning of the session, with participants randomly performing Session 1 (blue lines) and Session 2 (orange lines). In this study, Session 1 exhibited greater brain activity compared to Session 2, suggesting that purchasing gifts for others demands more mental effort from consumers.

We investigated the differences in decision-making depending on the purchase intentions in connection to brain activities. Fig 3 showed the task-related ΔHbO and ΔHbR signals. The ΔHbO and ΔHbR represented the average hemodynamic signals across all participants, comparing Session 1 (blue lines), where participants shopped for a red ginseng product as a gift for others, with Session 2 (orange lines), where participants shopped for a red ginseng product for self-consumptions in an online shopping mall environment.

Specifically, Fig 3A illustrated Sessions 1 and 2 based on the 15 channels of the prefrontal lobe located on the forehead with the averaged ΔHbO and ΔHbR of all participants. Session 1 showed higher brain activities than Session 2, indicating that buying gifts for others requires more cognitive effort from consumers. Furthermore, ΔHbO levels in Session 1 exhibited greater activation across most channels than in Session 2, with some channels showing overlapping activation levels between Sessions 1 and 2. Also, Fig 3B depicted the averaged ΔHbO and ΔHbR of all channels, which showed Session 1 and Session 2 together. Here, Session 1 shows higher brain activities than Session 2, which represented a more pronounced pattern than Fig 3A. These outcomes were closely linked to our mental workload and could evaluate human cognitive and mental responses.

## 4. Discussion

### 4.1. Impact of aesthetic packaging on purchase decisions

This study extends the existing literature [32,33] by providing empirical evidence on the impact of aesthetic packaging on consumer purchase decisions using fNIRS technology. Previous research has indicated that packaging significantly influences consumer perceptions and purchase intentions, particularly at the point of sale [21]. Our findings align with Baccarella et al., who noted that packaging elements like color and design play a crucial role in attracting consumers and differentiating products in the marketplace [34]. In our study, it was observed that the majority of participants exhibited increased cotical oxygen consumption during cognitive tasks, which is reflected in changes in the ∆HbO signals. It is consistent with the findings of Jöbsis et al. [35]. Additionally, the higher average HbO signals in the PFC during Session 1 (gift purchasing) suggest that consumers allocate more cognitive resources to gift decisions, reflecting greater emotional and social considerations. These results are also consistent with prior research [36]. It is known that stronger social interactions between others and the self, increase the average HbO signals.

Furthermore, several studies have shown that high levels of PFC activations were already demonstrated when participants made purchase decisions [21,37,38]. This is closely related to human mental workload and can be estimated based on hemodynamic responses. Likewise, we observed similar trends across all participants and all channels. This study shows that ΔHbO levels across most channels typically decrease, followed by a return to baseline after the completion of the session. Similar patterns of Session-induced negative hemodynamic responses (i.e., reductions in ΔHbO) have been observed in numerous prior studies on mental workload [39,40]. The reason is that the participants’ brains utilized increased oxygen levels during cognitive tasks [35].

Consequently, our research demonstrates that aesthetic packaging significantly influences consumer purchasing decisions, particularly for gifts, as evidenced by elevated HbO signals in the PFC during gift purchases, which indicate increased cognitive, emotional, and social engagement.

### 4.2. Neuromarketing insights for personalized marketing strategies

Our study utilizes neuromarketing techniques to provide detailed insights into consumer behavior, supporting the development of personalized marketing strategies. Previous studies using surveys have shown that consumers perceive aesthetically pleasing packaging as more valuable [41,42]. However, this approach has a limitation in that the results may vary depending on the subjective intentions of the participants. By utilizing fNIRS, we objectively measured brain activity, providing physiological evidence to support these findings. This methodology aligns with the findings of [20], which emphasize the importance of understanding unconscious consumer responses to enhance marketing effectiveness. Our findings suggest that marketing strategies should be tailored to both self-consumption and gift purchases, emphasizing functionality for personal use and visual appeal for gifting.

### 4.3. Advancing consumer behavior analysis with artificial Intelligence and neuromarketing

Incorporating insights from the field of neuromarketing, as highlighted in studies [43–45], these findings further validate the role of advanced methodologies, such as artificial neural networks and neuroimaing devices like fNIRS, in understanding consumer behavior. The cited studies highlight the predictive power of neuromarketing techniques in forecasting consumer responses, which is consistent with our approach of using fNIRS to objectively measure brain activity. By integrating these techniques, marketers can more accurately simulate and forecast consumer behavior, thereby enhancing the effectiveness of advertising campaigns.

### 4.4. Limitations and future research directions

Despite the valuable insights provided by this study, further investigation is needed to enhance the robustness and applicability of the findings. The homogeneous sample of healthy young adults and a limited sample size of 50 participants restrict the generalizability of the results. Future research should aim to include more diverse demographic groups to better represent the broader consumer population and improve the applicability of the findings to real-world contexts.

In addition, while this study primarily focused on the impact of aesthetic packaging, future research could broaden its scope to examine the influence of other sensory elements, such as texture and sound, on purchase decisions. Exploring these sensory dimensions could provide a more comprehensive understanding of how various packaging characteristics influence consumer behavior.

Longitudinal studies are also needed to explore how consumer preferences and neural responses evolve over time with repeated exposure to different packaging designs. These studies could offer valuable insights into the temporal dynamics of consumer behavior and further substantiate the current findings, contributing to the development of more effective neuro-data-driven marketing strategies.Moreover, integrating fNIRS with other neuroimaging tools, such as EEG or PET, as suggested by previous research [6], could yield a richer dataset and deeper insights into the neural mechanisms underlying consumer decision-making. Combining these methods with advanced machine learning techniques, as discussed in related studies, could enable large-scale predictions and simulations of consumer responses. This approach could pave the way for broader and more impactful applications of neuromarketing in the advertising industry.

## 5. Conclusion

This study provides significant insights into the effectiveness of neuromarketing techniques by utilizing fNIRS to analyze consumer behavior concerning Korean red ginseng products. These findings suggest that brain activity measured through fNIRS can offer valuable data on consumer preferences and purchasing decision-making processes. By measuring brain activity in the PFC, we identified distinct cognitive and emotional responses associated with purchasing decisions for self-consumption and gifting purposes. These findings underscore the importance of tailored packaging strategies in meeting diverse consumer needs and preferences.

Our research demonstrated a clear distinction in purchasing behavior when comparing sessions, highlighting the influence of branded packaging on consumer choices. The collected data highlight the importance of tailored marketing strategies, emphasizing that packaging for self-consumption and gifting should be distinctly designed to cater to different consumer needs.

Furthermore, this study reinforces the potential of fNIRS in predicting consumer purchasing behavior. It suggests that the fNIRS technique can be a powerful tool in the field of neuromarketing. By enhancing our understanding of the neural underpinnings of consumer behavior, businesses can better strategize their marketing efforts to align with consumer preferences, ultimately driving sales and customer satisfaction.

From a broader perspective, this research highlights the potential of integrating neuroscience with marketing to bridge the gap between consumer psychology and commercial strategies. The use of fNIRS offers a non-invasive and cost-effective method for studying unconscious consumer responses, providing objective data that complement traditional self-reported measures. This approach can be extended to various industries beyond health food sector, fostering the development of more personalized and effective marketing solutions.

Future research should address the limitations of this study, such as the relatively homogeneous sample and limited number of participants, by involving more diverse demographic groups. Furthermore, longitudinal studies could examine how repeated exposure to different packaging designs influences consumer preferences and neural responses over time. Investigating other sensory dimensions, such as texture and sound, could further enrich our understanding of how multi-sensory stimuli shape consumer decision-making.

Finally, combining fNIRS with other advanced neuroimaging technologies, such as EEG and PET, along with the integration of machine learning techniques, could enable more precise predictions and simulations of consumer behavior. These advancements have the potential to facilitate the widespread adoption of neuromarketing strategies, thereby enhancing their effectiveness in both academic research and practical business applications.

## Supporting information

S1 File Institutional Review Board(eng). (PDF)

S2 File Institutional Review Board(kor). (PDF)
