# Supplementary material for: Analysis of consumer purchase intentions using functional near-infrared spectroscopy(fNIRS): A neuromarketing study on the aesthetic packaging of Korean red ginseng products
Source: PLoS One. 2025 Jun 17;20(6):e0326213. doi: 10.1371/journal.pone.0326213 (PMC12173390; doi:10.1371/journal.pone.0326213)
Supplement: S1 File — (PDF) [file pone.0326213.s001.pdf]

Institutional Review Board
Wonkwang University

460 Iksan-daero, Iksan, Jeonbuk 54538, KOREA
Phone:82-63-850-7027 / FAX:82-63-850-5155

Institutional Review Board

Wonkwang University

Aug. 13, 2024

Research Title: Brain cognitive pattern analysis according to red ginseng purchase decision based on functional near-infrared spectroscopy

Researcher: Jaeyoung Shin
Affiliation: Department of Electronic Engineering, Wonkwang University

The this research was approved on the Institutional Review Board of Wonkwang University.

Approval Date: May. 22, 2024

Approval Period: May. 22, 2024 ~ May. 21, 2025

Approval Number: WKIRB-202405-HR-023

Sincerely,

Ji Sook Kang, RN, GNP, Ph.D.

Chairman
Institutional Review Board
Wonkwang University, Iksan, Korea
E-mail: jskang@wku.ac.kr
