# Supplementary material for: Analysis of consumer purchase intentions using functional near-infrared spectroscopy(fNIRS): A neuromarketing study on the aesthetic packaging of Korean red ginseng products
Source: PLoS One. 2025 Jun 17;20(6):e0326213. doi: 10.1371/journal.pone.0326213 (PMC12173390; doi:10.1371/journal.pone.0326213)
Supplement: S2 File — (PDF) [file pone.0326213.s002.pdf]

[원광대학교 생명윤리위원회]

WKU IRB
Wonkwang University Institutional Review Board

심의결과통보서

수 신 : 신 재 영 귀하

연구과제명 : 기능적 근적외선 분광법 기반 홍삼구매결정에 따른 뇌 인지적 패턴 분석

신청접수일 : 2024. 5. 14.

심의 일자	2024년 5월 22일
심의대상	☑ 연구계획서(신규) ☐ 연구계획서(보완)<br>☐ 연구계획서(변경) ☐ 승인된 연구계획서(지속 심의)<br>☑ 대상자동의서 ☐ 설문지, 질문지<br>☑ 모집광고 ☑ 증례(실험)기록지<br>☐ 기타 대상자에게 제공되는 문서 ☐ 중간보고서<br>☐ 종료(중지, 조기) 보고서 ☐ 결과보고서<br>☐ 기타 : ____________
심의 종류	☐ 정식심의 ☑ 신속심의
심의 결과	☑ 승인 ☐ 수정 후 승인 ☐ 보완 (☐ 신속 ☐ 정식)<br>☐ 반려 ☐ 승인된 연구의 중지 또는 보류
연구승인일	2024년 5월 22일
승인 유효기간	2024년 5월 22일 – 2025년 5월 21일
연구승인번호	WKIRB-202405-HR-023
지속심의주기	☐ 3개월 ☐ 6개월 ☑ 1년 ☐ 기타 (연구 종료 후 보고)
심의 의견	1. 성인을 대상으로 근적외선 측정장치를 이용해 시각적 자료에 대한 뇌 혈류 변화를 측정하는 연구로 침습적이지 않아 안전에 문제가 없다고 여겨지므로 연구 계획을 승인합니다.<br>2. 아래의 연구자 준수 사항을 지켜주시기 바랍니다.

* 연구자 준수사항

1. 위원회의 승인을 받은 연구계획서에 따라 연구를 수행해야 합니다.
2. 연구대상자로부터 동의를 획득하여야 하는 경우, 위원회의 승인을 받은 동의서 및 설명문을 사용하고, 강압이나 부당한 영향이 없는 자유로운 상태에서 충분한 설명에 근거하여 적법한 동의를 획득하여야 합니다.

3. 연구를 변경하고자 하는 경우 위원회의 승인을 받은 후 수행하여야 합니다. 단, 연구대상자를 보호하기 위해 불가피하게 연구를 변경하여 수행한 경우 즉시 해당 사항을 위원회에 보고하여 심의를 받아야 합니다.
4. 연구로 인해 연구대상자에게 예상하지 못한 중대한 위험이 발생하거나 발생시킬 우려가 있는 경우에는 지체 없이 연구를 일시중지 시키거나 조기종료 시키는 등의 필요한 조치를 취하고 즉시 위원회에 보고하여야 합니다.
5. 위원회가 연구에 대해 조사하거나 감독하는 경우 적극 협조하여야 하고, 기타 위원회가 요구하는 경우 연구와 관련된 사항에 대해 보고해야 합니다.
6. 승인 유효기간 만료 전에 중간보고를 하여 지속심의를 받아야 합니다.
7. 연구 종료 후 3개월 이내에 종료보고를 하여야 합니다.
8. 연구와 관련된 기록은 연구가 종료된 시점을 기준으로 최소 3년간 보관해야 합니다. 인체유래물 등 관리대장은 제공한 날부터 5년간 보관하여야 합니다. 기타 연구노트 등은 관련 법률에 따라 보관하여야 합니다.

2024년 5월 22일

원광대학교 생명윤리위원회 위원장 (인)
